# Corrosion inhibition of mild steel in 1 M HCl by pyrazolone-sulfonamide hybrids: synthesis, characterization, and evaluation

**DOI:** 10.1038/s41598-023-45659-2

**Published:** 2023-10-29

**Authors:** Ashraf M. Ashmawy, M. A. Mostafa, Abo-Bakr Kamal, Gomaa A. M. Ali, M. S. A. El-Gaby

**Affiliations:** 1 Chemistry Department, Faculty of Science, Al-Azhar University, Nasr City, Cairo, 11884 Egypt; 2 Chemistry Department, Faculty of Science, Al-Azhar University, Assiut, 71524 Egypt

**Keywords:** Electrochemistry, Chemistry

## Abstract

Carbon steel is widely used in the petroleum industry for pipelines, storage tanks, and equipment due to its mechanical properties, and strength. However, challenges such as environmental conditions and corrosive materials can affect its lifespan and require maintenance and repair. This work aimed to prepare pyrazalone-sulfonamide hybrids, and confirmed by mass spectra, FTIR, ^1^H-NMR, and ^13^C-NMR. These compounds were examined as mild steel corrosion inhibitors in 1 M HCl solutions at 298–323 K using the gravimetric technique, electrochemical measurements, scanning electronic microscope analysis, and quantum chemical calculations. The values of inhibitory efficiency identified by electrochemical and non-electrochemical techniques exhibit good agreement. At various temperatures and in the 50 to 500 ppm concentration range. During the adsorption process, these substances connect to the Langmuir adsorption isotherm. Some adsorption isotherm and kinetic parameters have been developed and discussed. The metal surface had a thin inhibitory protective layer, according to investigations using energy dispersive X-ray spectroscopy (EDX) and scanning electron microscope (SEM). These findings demonstrated the potential of pyrazolone-sulfonamide as effective organic corrosion inhibitors for carbon steel.

## Introduction

Corrosion is an indication of how reactive the majority of industrial materials are to the environment in which they are used^1–3^. Iron continues to be the most used material in engineering. Due to its inexpensive cost and flexible mechanical properties, iron is the backbone material. Steel is still the most often used material for usage in transmission pipes, flow lines, plates, sheets, tubing, and structural components in the oil and gas industry^4,5^. Hydrochloric acid is one of the most often used acidic substances for acid descaling, industrial cleaning, acid pickling, and cleaning metal surfaces to avoid corrosion damage^6^. Localized corrosion is frequently brought on by aggressive ions known as chlorides (Cl^−^). Their presence in solutions has two complementary effects: on the one hand, their local concentration makes the medium more acidic, and on the other hand, salinity alters the conductivity of the aqueous medium. In brackish or potable water, steel naturally corrodes^7^. The organic molecules inhibit corrosion by adhering to the metal’s surface. The two fundamental forms of interaction that may be employed to describe these adsorption processes are physical adsorption and chemisorption^8^. The nature and charge of the metal, the chemical composition of the organic product, and the kind of electrolyte all have an impact on these two forms of adsorption^9,10^. The use of organic compounds as corrosion inhibitors is still a study topic that is being explored, if our conclusions are based on the number of publications that are published each year. Moreover, it has been discovered that heterocyclic compounds containing heteroatoms like nitrogen, sulfur, and oxygen are the most efficient defenses against steel corrosion under acidic environments^11–14^. Moreover, studies into the anticorrosive properties of heterocyclic triazoles, thiadiazol, pyrazole, and other substances have thus far yielded good results. This area of research is essential because these compounds can replace potentially harmful inhibitors that are regarded to be unacceptable for the environment^15–23^.

The process of connecting or fusing two different chemical entities results in new hybrid moieties and is known as molecular hybridization. It is expected that the pharmacological activity of the hybrid compounds will be additive or synergistic. The two parts were selected based on their previously known bios. Since several of these hybrid derivatives were more effective and had a lower toxicity profile than their parent chemicals, they were widely used^24,25^.

**Figure 1:**
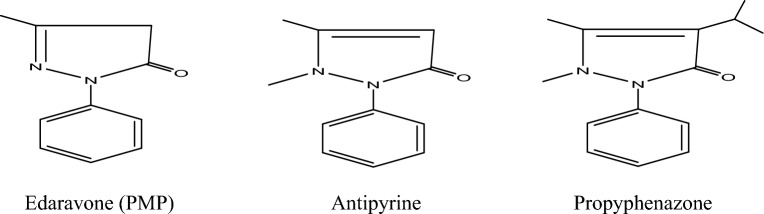
Phenyl-3-methyl-2-pyrazol-5-one, known as PMP or Edaravone and the related pharmaceutical products antipyrine (phenazone) and propyphenazone.

The pyrazoline derivatives are an intriguing group of heterocyclic compounds because of the discovery of fresh lead compounds with a wide range of biological characteristics and the significant role they play in drug creation. They stand out for their adaptability in the synthetic process as well^26^. The phenyl-pyrazolone unit is found in a variety of compounds with potential as medicines. With especial, 1-phenyl-3-methyl-5-pyrazolone (PMP), also known as edaravone Fig. 1, is a bicyclic chemical structure present in a variety of medications, most notably edaravone but also antipyrine (phenazone) and propyphenazone as examples Fig. 1. Essentially, the 1-phenyl-3-methyl-5-pyrazolone molecule will resemble the PMP reagent a biochemists use to analyze carbohydrates. For those in the medical field, the drug edaravone, which is used to treat acute stroke harm and amyotrophic lateral sclerosis, will mostly have the same structure (ALS). The structure can be viewed as an intriguing framework that medicinal chemists can use to build new drugs, especially ones that are antioxidative and/or anticancer^27^. One of the most important types of antibacterial chemicals is sulfonamide derivatives^28^. Many compounds containing sulfonamide groups also exhibit additional essential biological traits, such as anticancer activities^29^, antidiabetic type 2^30^, and antifungal activities^31^. Nowadays, pyrazoline and sulfonamide scaffold hybridization has been investigated^26^. Sayed et al. reported on the synthesis and anticancer evaluations of pyrazolone-sulfonamide hybrids as against HepG2, HCT-116, and MCF-7 cancer cell lines using the pharmacophore hybridization approach^32^. Yamali et al. reported the synthesis of new pyrazoline-sulfonamide hybrids and in vitro pharmacological assessment as multi-target agents for the inhibition of acetyl cholinesterase and carbonic anhydrase I and II enzymes^33^. The molecular structure, electrical structure, and reactivity of heterocyclic organic compounds have been determined using the density functional theory (DFT), which has also been used to light the process of corrosion inhibition^34,35^.

**Figure 2:**
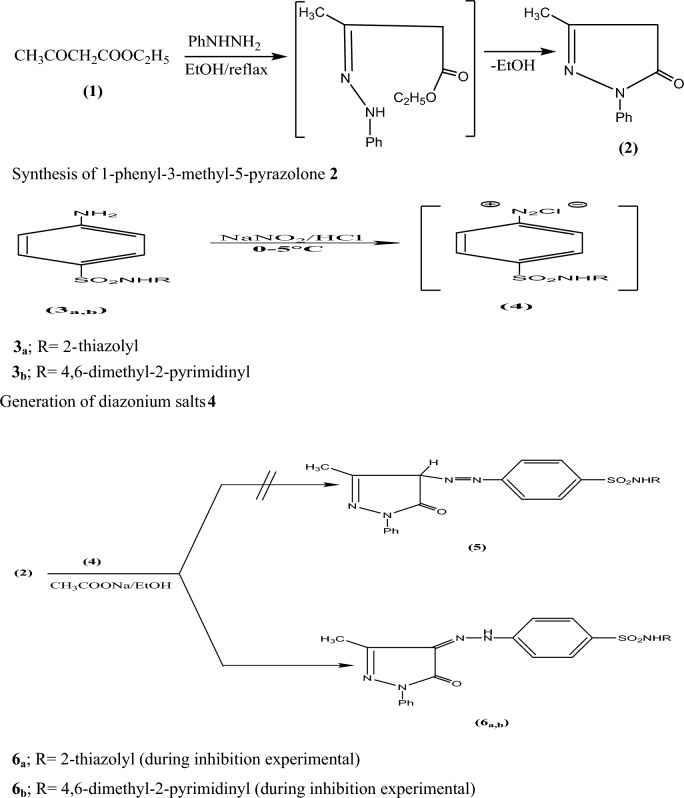
Scheme for synthesis of pyrazolone-sulfonamide hydrids **6a, 6b**.

In this study, we developed pyrazalone-sulfonamide hybrids by combining these two molecular parts into a single framework, in which the sulfonamide fragment was attached to the pyrazalone through a variety of linkers as shown in Fig. 2, and we tested these hybrids as mild steel corrosion inhibitors in 1 M HCl. Electrochemical frequency modulation, electrochemical impedance spectroscopy (EIS), and potentiodynamic polarization were a few of the techniques employed to evaluate the inhibitory impact (PDP).

## Experimental procedure

### Materials instruments

The substances were high purity, analytical reagent grade compounds. The British Drug House provided the organic solvents**.** A Gallenkemp melting point detector was used to find melting points (OC, uncorrected) in open capillaries (Sanyo Gallenkemp, Southborough, UK). IR spectra (KBr discs) were obtained using Fourier transform infrared spectroscopy (FTIR, plus 460 or Pye Unicam SP-1000 spectrophotometer, Pye Unicam, Cambridge, UK). ^1^HNMR spectra (DMSO-d6) were collected using a Bruker spectrophotometer (400 MHz for ^1^HNMR and 100 MHz for ^13^CNMR). Tetraethyl silane is used as an internal standard, while DMSO-d6 is used as the solvent. Chemical shifts are displayed in ppm. MS electrodes with the compositions and pre-treatment information described in our prior study were purchased from the AL-EZZ firm in Alexandria, Egypt. The 1 M HCl solution that is corrosive was created by diluting 37% hydrochloric acid^36^. The morphological investigation of the MS was conducted using the scanning electron microscope (SEM, JEOL, JSM5400LV).TLC on silica gel 60 F254 plates, visualization with UV light (254 nm), monitoring the reaction progress, and assessing the purity of synthesized compounds.

### Inhibitor synthesis

#### Synthesis of 1-phenyl-3-methyl-5-pyrazolone2

Ethyl acetoacetate 1 (0.01 mol) and phenyl hydrazine (0.01 mol) were dissolved in ethanol (20 ml) and refluxed for one hour before the solvent was evaporated. The equivalent pyrazole 2 was produced as light yellow crystals when the solid residue crystallized from ethanol (Lit.mp 126–128 °C)^37^.

#### Synthesis of 4-(2-(3-methyl-5-oxo-1-phenyl-1H-pyrazol-4(5H)-ylidene) hydrazinyl)-N-(thiazol-2-yl)benzenesulfonamide 6a

**Figure 3:**
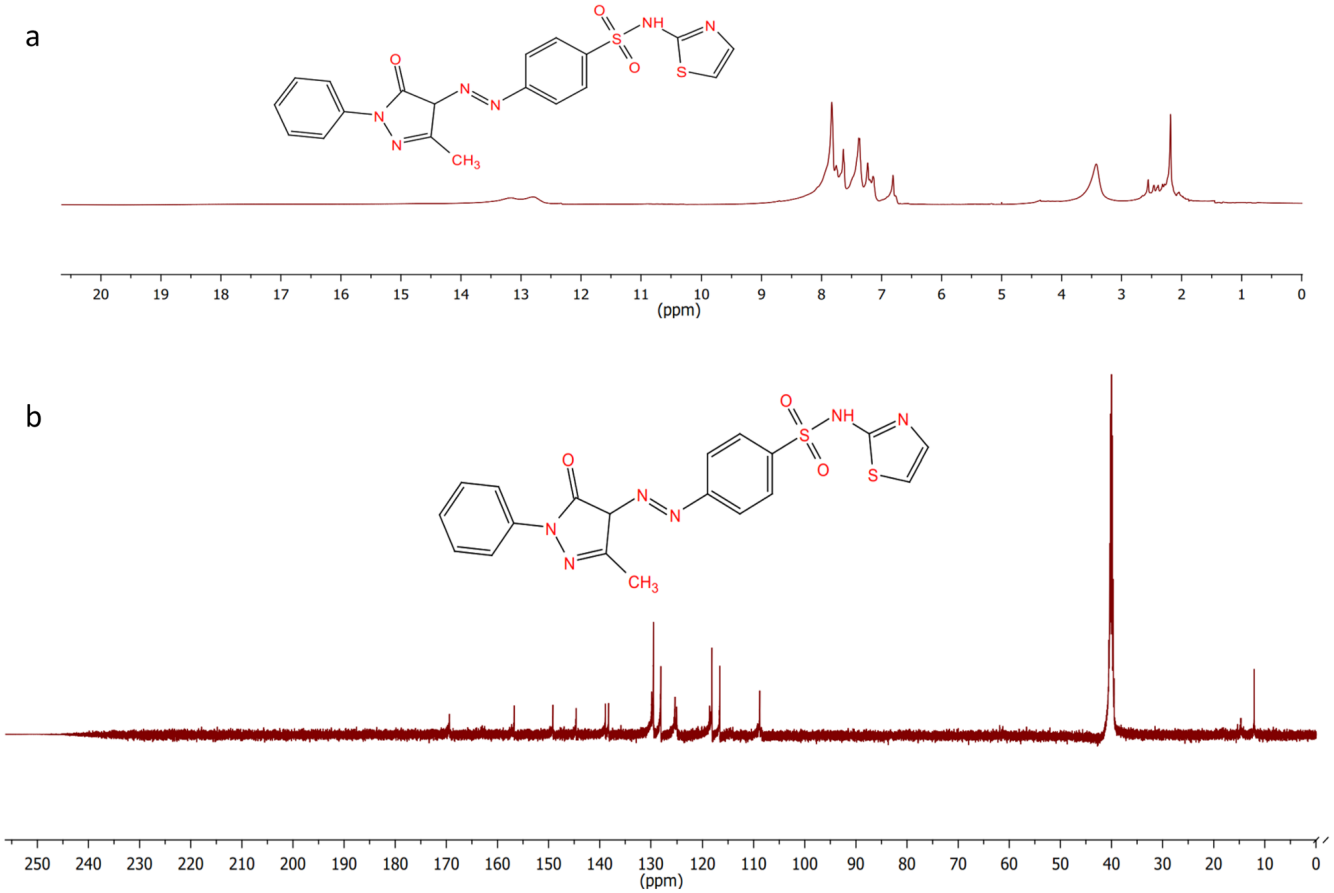
(**a**) ^1^H NMR and (**b**) ^13^C NMR spectrum of the synthesized compound 6a.

In 50 mL of water, 0.1 mol of sulfathiazole 3a was suspended. Then under stirring conditions, 10 mL of 36.5% HCl was added to this solution. The mixture was progressively heated to 60 °C until transparent. An ice bath was used to cool the solution from 0 to 5 °C. The addition of a sodium nitrite solution (0.5 g in 5 mL water) was stirred for 5 min. 1-Phenyl-3-methyl-5-pyrazolone 2 (0.1 mol) was dissolved in 20 mL of (95%) ethanol and kept at 0 to 5 °C. With sodium acetate. the diazonium salt solution was added for 10–15 min while being continuously slowly stirring to get a pure product of 4-(2-(3-methyl-5-oxo-1-phenyl-1*H*-pyrazol-4(5*H*)-ylidene) hydrazinyl)-*N*-(thiazol-2-yl)benzenesulfonamide **6a**, The precipitate that was formed was filtered, dried, and then recrystallized in 85% aqueous ethanol. solid colour of red.m.p. 145–146 °C, yield 92%. IR (KBr, cm^–1^): 3100(NH), 2911(CH-alph), 1689 (C=O), 1595 (N=N), 1362, 1143 (SO_2_); ^1^HNMR (400 MHz, DMSO-*d*_*6*_) δ: 2.25 (s, 3H, CH_3_), 6.74, 7.21 (2s, 2H, thiazole-H), 7.89 (d, 2H, AB-system), 8.01 (d, 2H, AB-system), 7.45–7.73 (m, 5H, arom-H), 11.80, 13.20 (2s, 2H, 2NH changeable with D_2_O). ^13^CNMR (DMSO-*d*_6_, 75 MHz) δppm): 12.09 (CH_3_), 108.81, 116.62, 118.61, 125.39, 128.01 (C=N), 129.85, 138.25, 138.90, 144.62, 149.15, 156.73 (C=O), 169.37 (C=N). Anal. Calcd for: C_19_H_16_N_6_O_3_S_2_ (440): C, 42.70; H, 3.94; N, 24.90; S, 11.40. Found: C, 42.60; H, 3.90; N, 24.80; S, 11.50 (see Fig. 3, Fig. S1).

#### Synthesis of *N*-(4,6-dimethylpyrimidin-2-yl)-4-(2-(3-methyl-5-oxo-1-phenyl-1*H*-pyrazol-4(5*H*)-ylidene)hydrazinyl)benzenesulfonamide 6b

This compound was produced from sulfadimidine. **3b** (0.1 mol) and 1-Phenyl-3-methyl-5-pyrazolone** 2** (0.1 mol) in the same way that was explained for the preparation of **6a**.

**Figure 4:**
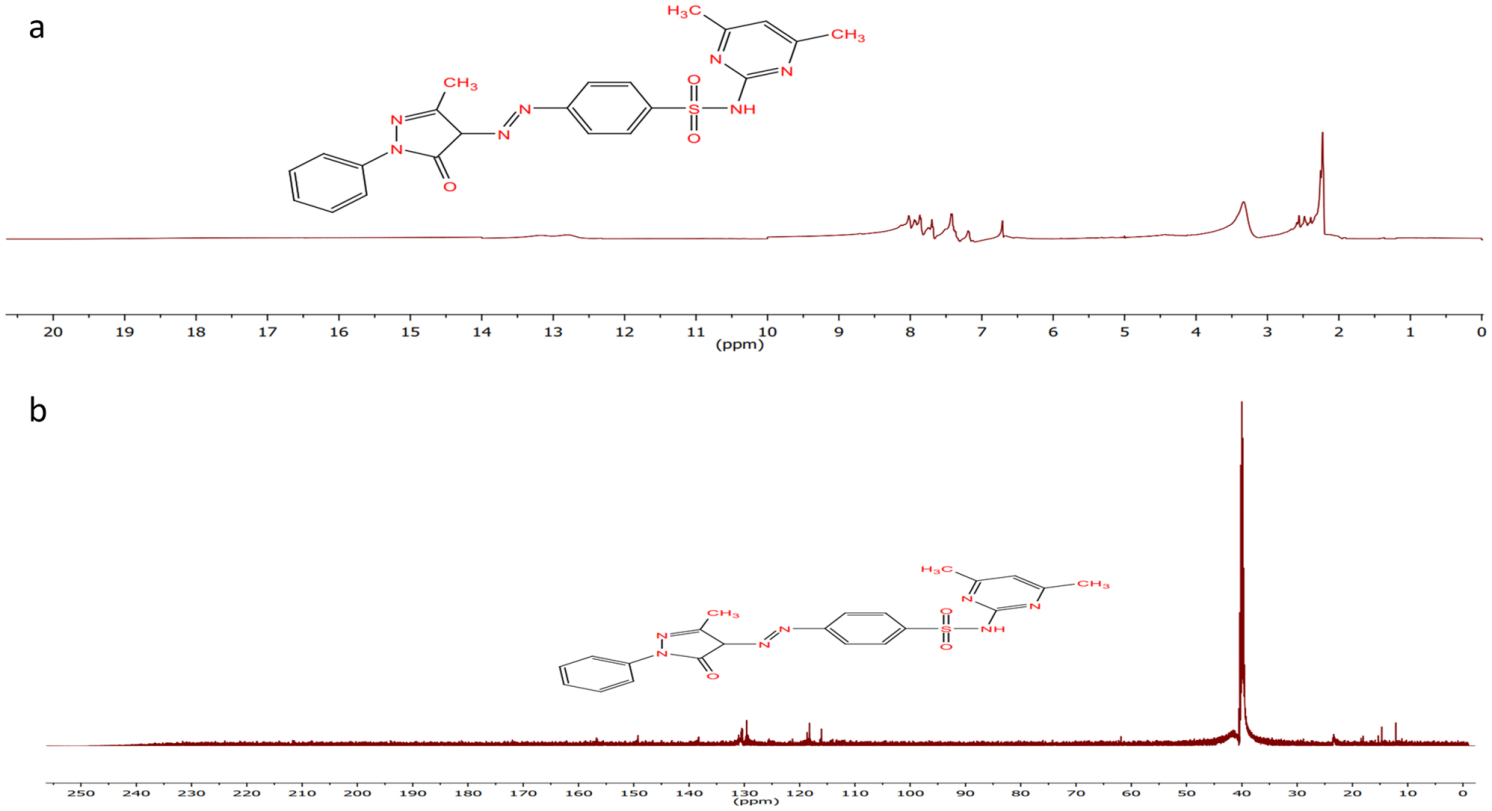
(**a**) ^1^H NMR and (**b**) ^13^C NMR spectrum of the synthesized compound 6b.

Orange color solid. m.p. 165–166 °C, yield 92%. IR (KBr, cm^–1^): 323 3(NH), 3020 (CH-arm), 2925 (CH-alph), 1693 (C=O), 1597 (N=N), 1344, 1153 (SO_2_); ^1^HNMR (400 MHz, DMSO-*d*_6_) δ: 2.29 (s, 3H, CH_3_), 2.42 (s, 6H, 2CH_3_), 6.79 (s, 1H, pyrimidine-H), 7.24–7.90 (m, 9H, arom-H), 12.80, 13.30 (2s, 2H, 2NH exchangeable with D_2_O). ^13^CNMR (DMSO-*d*_6_, 75 MHz) δ (ppm): 12.17 (CH_3_-pyrazoline), 23.48 (CH_3_-yrimidine), 116.07, 118.39, 129.56, 130.47 (C=N), 137.81, 138.26, 144.98, 147.79 (C=N), 149.26, 154.15, 156.63 (C=O), 162.39, 162.90 (C=N), 167.90 (C=N). Anal. Calcd for: C_22_H_21_N_7_O_3_S (463): C, 57.01; H, 4.57; N, 21.15; S, 6.92. Found: C, 57.00; H, 4.50; N, 21.10; S, 6.90 (see Fig. 4).

### Preparation of metal electrode

Mild steel (MS) of the following chemical structure (wt%): C—0.093, P—0.014, Si—0.011, Mn—0.853, Cr—0.025, Cu—0.012, Al—0.032, Ni—0.013 and Fe—In the experiments, balancing was used^38^.

### Gravimetric technique

Experiments were conducted on MS in 1 M HCl acid (with and without inhibitors) to measure the weight loss. The MS sheets utilized have dimensions of 5 × 1 × 0.1 cm. The sheets were polished to variable degrees to achieve different granulation grades (SiC 1200 and 1400). The test solution was placed in a 250 mL glass vessel for each run. A clean weight was placed in an upright position and filled to the top of the jar. The plates are dried by air after being warmed. Millilitter of the test solution rinsed and dried in ethanol. A clean weight sample of mild steel was placed in an inclined position in the jar. The specimen was removed and cleaned with distilled water to remove abrasion products after spending a day immersed in 1 M HCl (uninhibited and inhibited) with the addition of different dosages of inhibitors. The plates are then heated air dried. ethanol-washed, dried, and weighed. Thermostat-controlled experiments were conducted at constant temperatures of 298, 303, 313, and 323 K in freshly produced solutions. The corrosion rate (CR) was calculated using the weight loss^39^.

### Electrochemical investigations

The tests were performed using an electrochemical cell with three electrodes that has a saturated calomel electrode (SCE) as the reference electrode, a platinum wire as the counter electrode, and MS with a surface area of 1 cm^2^. In this study, electrochemical impedance spectroscopy, electrochemical frequency modulation, and electrochemical potentiodynamic polarization approaches have all been measured in turn. Electrochemical impedance spectroscopy (EIS) was performed using an electrode that had been submerged in the test for an hour. The EIS measurements were made at open-circuit voltage (OCV) with a small alternating voltage perturbation (10 mV) applied to the cell over the frequency range of 100 kHz to 20 mHz at 298 K. Second, electrochemical frequency modulation (EFM) was carried out using two frequencies of 2 and 5 Hz and an AC amplitude of 10 mV. Potentiodynamic polarization was also detected for anodic and cathodic polarization at a scan rate of 5 mV s^−1^. With the use of the Gamry 3000 potentiostat/galvanostat/ZRA and Echem Analyst 7 software, all of these methods were measured^40^.

### Quantum chemical calculations

Quantum chemical calculations of many parameters were calculated using the (DFT) method. DFT using Becke’s three parameter exchange functional (B3LYP) at the 6-311G (d,p) basis set^41^ drawn in the GaussView 5.0 with Gaussian 09W program package^42^. Quantum chemical parameters such as the highest occupied molecular orbital (E_HOMO_), the lowest unoccupied molecular orbital (E_LUMO_), the energy gap (ΔE), the electron affinity EA, the ionization potential IP, the electronegativity (χ), the global hardness (η), the softness (σ), the electrophilicity index (ω), dipole moment (DM), total energy (TE), total negative charges and the fraction of electrons transferred ΔN were calculated by the following Eqs. (1)–(6)^43–46^.

1 $$\Delta {\text{E}} = {\text{E}}_{{{\text{LUMO}}}} - {\text{E}}_{\text{HOMO,}}$$

2 $$\mathrm{A}=-\mathrm{E}_\mathrm{LUMO},$$

3 $$\mathrm{I}=-\mathrm{E_{HOMO}},$$

4 $$\upeta =\frac{ I-A}{2},$$

5 $$\upsigma =\frac{1}{\upeta },$$

6 $$\mathrm{\Delta N}= \frac{[\mathrm{\chi_{Fe}}-\mathrm{\chi_{inh}}]}{2[\mathrm{\eta_{Fe}}+\mathrm{\eta_{inh}}]},$$

where χ_Fe_ ≈ 7 eV^41^ is taken for iron and η_Fe_ = 0 is taken, assuming that the ionization potential, I, equal the electron affinity, A, for bulk metals where χ_Fe_ and η_Fe_ are 7 and 0 respectively^42^.

## Results and discussion

### Characterization of the inhibitor

In the present work, the intermediate 3-methyl-I-phenyl-5-pyrazolone **(2)**, named Edaravone (Radicava), was obtained quantitatively via the *Knorr* condensation reaction of ethyl acetoacetate **1** with phenyl hydrazine^37^. The formation of compound **2** is assumed via nucleophilic attack of the least hindered nitrogen atom of phenyl hydrazine on the ketone moiety of ethyl acetoacetate followed by intramolecular cyclization involving the ester and the second nitrogen atom of phenyl hydrazine^37^.

The reaction of 3-methyl-I-phenyl-5-pyrazolone **2** withdiazonium chloride of sulfathiazole **3a** gave 4-(2-(3-methyl-5-oxo-1-phenyl-1*H*-pyrazol-4(5*H*)-ylidene)hydrazinyl)-*N*-(thiazol-2-yl) benzenesulfonamide **6a** rather than the other possible structure **5**, Fig. 2. The synthesis involved initial diazotization of sulfathiazole **3a** with sodium nitrite in hydrochloride solution to afford the diazonium salt **4**, followed by coupling with a molar equivalent of the appropriate pyrazolone **2** in ethanol in the presence of sodium acetate at room temperature. The structure of compound **6a** was established based on its analytical and spectral data. The infrared spectrum of the compound **6a** showed the characteristic absorption band at 3100 cm^−1^ for the NH stretching: 1689 cm^−1^ for the C=O stretching and 1595 cm^−1^ for the N=N stretching vibrations (Fig. S2). The ^1^HNMR spectrum of compound **6a** (DMSO-*d*_6_) declared the lack of singlet characteristic for methine proton and showed a merged singlet of methyl protons attached to the 3rd position of the pyrazole ring at δ 2.25 ppm. In the aromatic region a multiplet was obtained at δ 7.45–7.73 ppm indicating the presence of five phenyl protons and two doublets at δ 7.89, 8.01 ppm assigned to the AB-system of benzene sulfonamide moiety in addition to the presence of two downfield signals at δ 11.80, 13.20 ppm assigned to the NH protons. ^13^CNMR spectral data of the compound showed a methyl group carbon at δ 12.09 and a carbonyl carbon downfield at δ 156.73. The other peaks of carbon were observed at δ 108.81, 116.62, 118.61, 125.39, 128.01 (C=N), 129.85, 138.25, 138.90, 144.62, 149.15, 169.37 (C=N) confirming the presence of nineteen carbons in the compound. The structure of compound **6a** was further confirmed by mass spectral data which showed molecular ion peak M^+^ at m/z 440 corresponding to molecular formula C_19_H_16_N_6_O_3_S_2_. The coupling reactions involved that initially generate strong ^+^N_2_ electrophile from amine then finally react and coupled at active methylene group in pyrazolone **2** to produce compound **6**, Fig. 2. In a similar manner, compound** 2** reacted with diazonium chloride of sulfadimidine **3b** at room temperature and afforded the *N*-(4,6-dimethyl pyrimidin-2-yl)-4-(2-(3-methyl-5-oxo-1-phenyl-1*H*-pyrazol-4(5*H*)ylidene)hydrazinyl)benzenesulfonamide **6b** in a good yield**.** The structure of the isolated product was confirmed because of its elemental analysis and spectral data. The infrared spectrum of compound **6b** showed strong bands at 1693 cm^−1^ which were due to carbonyl stretching of pyrazolone moiety. The compound showed bands at 3020 cm^−1^ and 2925 cm^−1^ which are due to aromatic C–H stretching and stretching aliphatic C–H vibration respectively. As shown in Fig. S2, bands observed at 1579, 1348, and 1153 cm^−1^ are assignable for N=N and SO_2_ groups, respectively. The ^1^H NMR spectrum of compound **6b** showed two singlets at δ 2.29 and 2.42 ppm indicating the presence of methyl groups at pyrazoline and pyrimidine moieties, respectively. The singlet signal observed at δ 6.79 ppm assigned to the methine proton present at 5th position of pyrimidine ring. A multiplet was observed in the aromatic region at δ 7.24–7.90 ppm indicating the presence of protons of two aromatic rings and in addition to the presence of two downfield signals at δ 12.80, 13.30 ppm assigned to the NH protons. The ^13^C-NMR spectrum of compound **6b** showed signals at δ 12.17, 23.48, 116.07, 118.39, 129.56, 130.47 (C=N), 137.81, 138.26, 144.98, 147.79 (C=N), 149.26, 154.15, 156.63, 162.39, 162.90 (C=N), 167.90 (C=N) corresponding to twenty-two different type of carbon atoms present in the compound. The most downfield signal appeared at δ 156.63 can be assigned to the carbonyl carbon of group in pyrazoline nucleus. The signals appeared at δ 12.17.84 and 23.48 can be assigned to methyl carbon of pyrazoline and pyrimidine, respectively.

### Gravimetric experiments

#### Effect of inhibitor concentrations on the corrosion rate

A first step in the investigation of how to stop a metal from corroding in an electrolytic solution is to evaluate mass loss. This approach has the benefit of being easy to use and requiring little equipment. In 1 M HCl, the mild steel samples are submerged, both with and without the addition of various quantities of (6a and 6b). Utilizing a thermostatic bath, efficiency inhibitory activity is assessed after 1 day of immersion at 298, 303, 313, and 323 K. The indicated inhibitory effectiveness value is the average of three experiments performed for each concentration under identical conditions according to the following relations (7) and (8):

7 $$\mathrm{CR}=3\frac{\mathrm{K}\times \mathrm{\Delta W}}{\mathrm{A}\times \mathrm{t}\times \mathrm{d}},$$

where K is a constant (8.76 × 10^4^), ∆W is the loss of weight after corrosion (mg), A is the metal surface area (cm^2^); t is the immersion time (h), d is the metal density (g/cm^3^).

The inhibition efficiency (IE_WL_, %) and surface coverage (θ) of inhibitors were calculated using the following expression^46^:

8 $${\mathrm{IE}}_{\mathrm{WL}}=\uptheta \times 100=\frac{{\mathrm{CR}}_{\mathrm{un }} - {\mathrm{CR}}_{\mathrm{in}}}{{\mathrm{CR}}_{\mathrm{blank }}}\times 100,$$

where CR_un_ and CR_in_ are the corrosion rates of mild steel due to the dissolution in 1 M HCl in the absence and the presence of a definite concentration of inhibitor, respectively.

**Figure 5:**
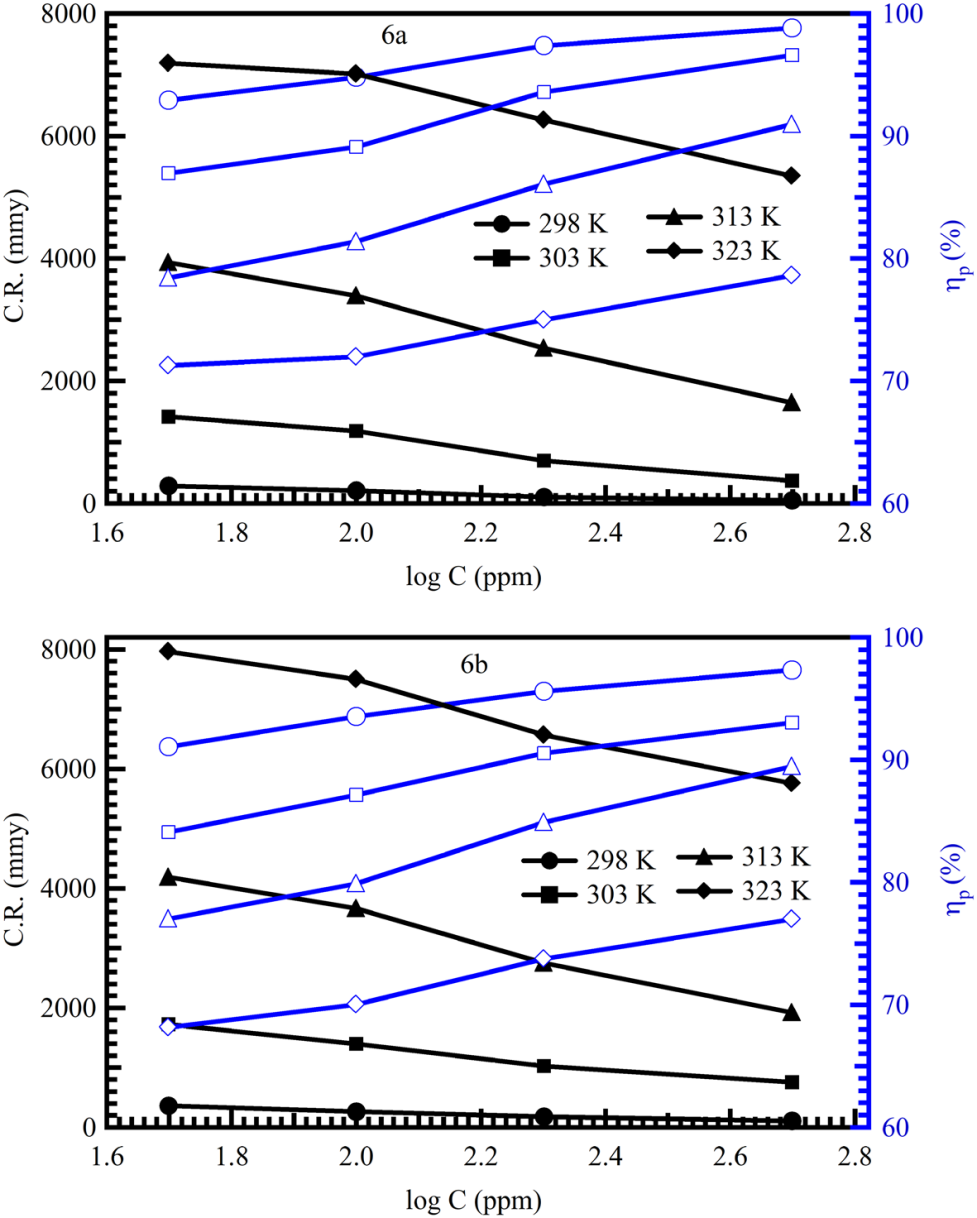
Relationship between corrosion rate (CR) and inhibition efficiency (ηp) with inhibitors concentrations at different temperatures of inhibitors in 1 M HCl.

Table 1 displays the MS corrosion characteristics in 1 M HCl with and without various amounts of (6a and 6b) at 298 K. The calculated values of corrosion rate, inhibition efficiency (IE percent), and the extent of surface coverage for MS dissolution in 1 M HCl in the absence and presence of the two inhibitors of (6a and 6b) indicated their ability to prevent corrosion of MS in 1 M HCl solutions, where the corrosion rate is concentration dependent. It is clear from Table 1 that at all doses utilized in this investigation, (6a and 6b) suppress mild steel corrosion in 1 M HCl solutions. The corrosion rate is shown in Fig. 5 to continuously decrease with increasing inhibitor concentration. Corrosion rate values for mild steel also decrease as inhibitor concentration rises, whereas (IE percent) values for (6a and 6b) rise as concentration rises, reaching maximum values at (95.17 and 94.02 percent) at 500 ppm, respectively. Adsorption on the metal surface can be used to explain the (IE percent) prevention of mild steel corrosion. The interaction between the metal surface and the lone pairs of electrons on the nitrogen and sulfur atoms of the inhibitor can cause these compounds to be adsorbed. The iron atom’s unoccupied low energy orbitals help to assist this reaction^47,48^. It was shown also that the corrosion rate depended on molecular weight. As molecule weight increases the inhibition efficiency increases^49^.

**Table 1:** Wight loss parameters of 6a and 6b as inhibitors on the corrosion of mild steel in 1 M HCl for 24 h immersion period.

Temperature	Conc. of inhibitors (ppm)	∆W (g)	C.R (mmpy)	I.E (%)	Surface coverage (θ)
298 K	Blank	0.02091	4051.0350	–	–
	6a				
	50	0.00198	383.5987	90.5308	0.905
	100	0.00179	346.7887	91.4395	0.914
	200	0.00155	300.2919	92.5873	0.926
	500	0.00125	242.1709	94.0220	0.940
	6b				
	50	0.00187	362.2877	91.0569	0.911
	100	0.00135	261.5446	93.5438	0.935
	200	0.00120	232.4841	94.2611	0.943
	500	0.00101	195.6741	95.1698	0.952

Table 1 and Figure 5 provide evidence that, for MS at all investigated temperatures, (6a and 6b) are very effective corrosion inhibitors. Since it was anticipated that (6a and 6b) would desorb at higher temperatures and offer lesser inhibitory efficiency, this result caught everyone off guard. However, it appears that during the entire temperature range under study, the molecules are stable on the MS surface. This is a significant finding from a practical standpoint since a wider range of industrial processes that take place at somewhat higher temperatures can now use these inhibitors^45^.

#### Adsorption consideration

**Figure 6:**
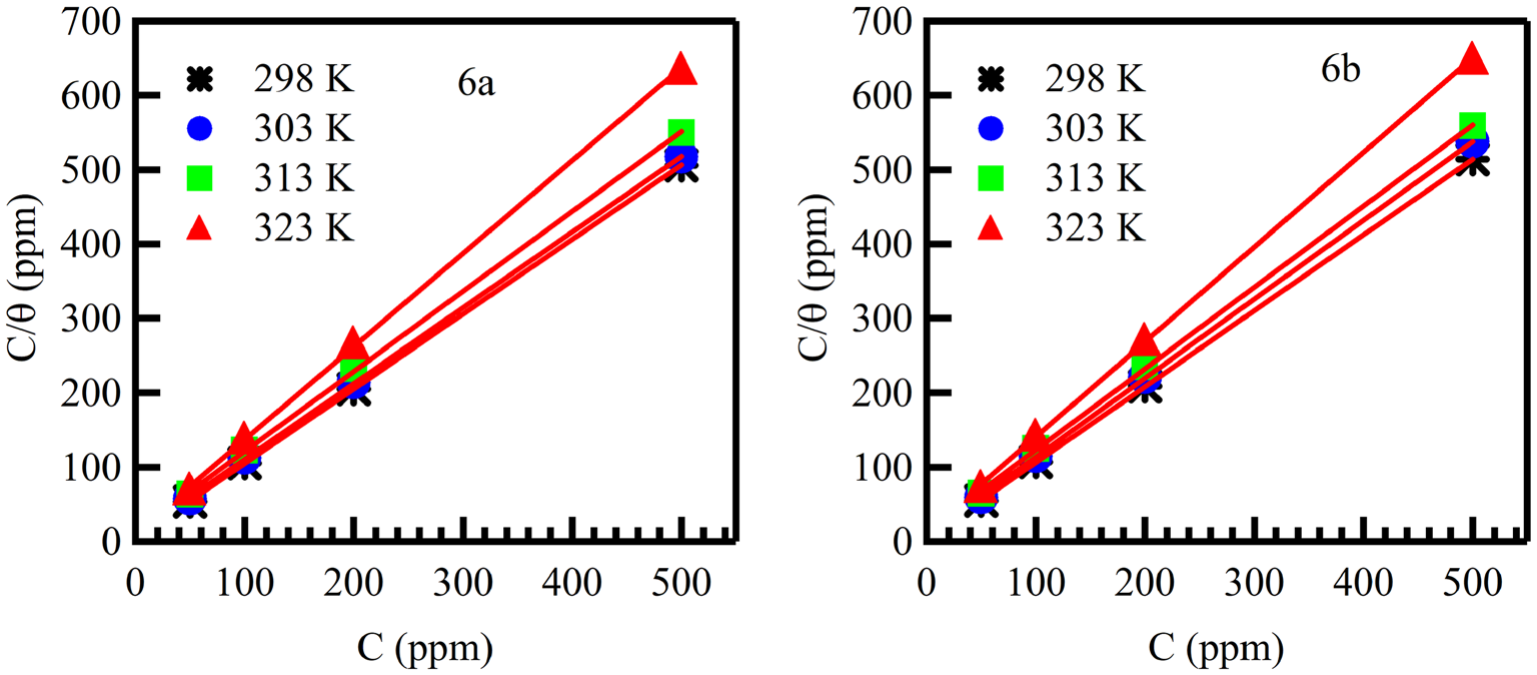
Langmuir adsorption plots for mild steel in 1 M HCl solutions at the presence of different concentrations of the inhibitors at different temperatures.

Figure 6 show (plot of C/θ against C) gives straight lines with slope equal or nearly equal to 1.00 for four temperatures (298, 303, 313 and 323 K) and intercepts provide the calculation of the values of K_ads_ are given in Table 2. The obtained results indicate that the adsorption of compounds under consideration on mild steel/acidic solution interface follows the Langmuir adsorption isotherm according to Eqs. (9) and (10).

**Table 2:** Adsorption isotherm parameters of the mild steel electrode surface in 1 M HCl containing different concentrations of the 6a and 6b at different temperatures.

Inhibitor name	Temp. K	K_ads_ *M*^−1^	∆G_ads_ kJ mol^−1^
6a	298	0.225	− 30.524
	303	0.114	− 29.318
	313	0.072	− 29.096
	323	0.083	− 30.388
6b	298	0.211	− 30.357
	303	0.127	− 29.583
	313	0.071	− 29.058
	323	0.075	− 30.123

9 $$\frac{C }{\theta }=\frac{1 }{{K}_{ads}}+C,$$

10 $${\mathrm{K}}_{\mathrm{ads}}=\frac{1 }{55.5}=\mathrm{exp}\left(\frac{\Delta {\mathrm{G}}_{ads}^{^\circ }}{\mathrm{RT }}\right).$$

As can be seen from Table 2, the addition of the inhibitors causes negative values of ∆G°_ads_, indicating that the adsorption of studied sulfonamide derivatives is a spontaneous process^50^ According to widespread consensus, adsorption types can be classified as physisorption for values of Goads up to − 20 kJ mol^−1^, and the inhibition is caused by electrostatic interactions between charged molecules and the charged metal. A covalent link is formed between the inhibitor molecules and the metal surface when the values are around − 40 kJ mol^−1^ or less, and this process is known as chemisorption^51,52^. According to Table 2, where the values of ∆Gº_ads_ in our experiments vary from − 29.06 to − 30.52 kJ mol^−1^, the adsorption of these sulfonamide derivatives is thought to involve two different types of interaction: chemisorption and physisorption^53^.

##### Effect of temperature

Temperature is one of the factors that can modify both the behavior of inhibitors and substrates in each aggressive medium. The organic compounds can dissolve more easily when the temperature increases. The increase in temperature can thus cause a weakening of the corrosion resistance of the metals^54^. To examine the influence of this parameter on the inhibitory efficiency of (6a and 6b), we conducted Wight loss measurements for temperatures equal to 298, 303, 313 and 323 K. The results obtained after 1 day of immersion time are summarized in Table 3. Table 3 shows that CR in 1 M HCl increases with increasing temperature. For all inhibitor concentrations, CR also increases with temperature, but takes lower values at high inhibitor concentrations. This allows us to observe that the (IE%) increases when the temperature decreases. This evolution is, however, more marked for the highest concentrations of the inhibitor.

**Table 3:** Activation parameters values for mild steel in 1 M HCl in the absence and presence of different concentrations of the studied 6a and 6b at different temperatures.

Inhibitor	Conc. of inhibitor (ppm)	Ea (kJ mol^−1^)	∆H* (kJ mol^−1^)	∆S* (kJ mol^−1^ *K*^−1^)
Blank	0.00	− 53.63	− 51.05	− 11.42
6a	50	− 96.51	− 93.94	− 119.37
	100	− 104.49	− 101.91	− 145.74
	200	− 122.64	− 120.06	− 201.12
	500	− 142.35	− 139.76	− 260.71
6b	50	− 91.60	− 89.02	− 106.68
	100	− 99.61	− 97.02	− 131.00
	200	− 106.83	− 104.24	− 152.06
	500	− 116.04	− 113.63	− 179.75

Using the Arrhenius Eq. (11), the activation energy (Ea) for the corrosion process was obtained as follows^55^.

11 $${\text{log}}\, C.R.= \frac{-{E}_{a}}{2.303RT}+{\text{log}} A.$$

R is the gas constant (8.31 J/mol/K), A is the Arrhenius pre-exponential factor, and T is the temperature.

**Figure 7:**
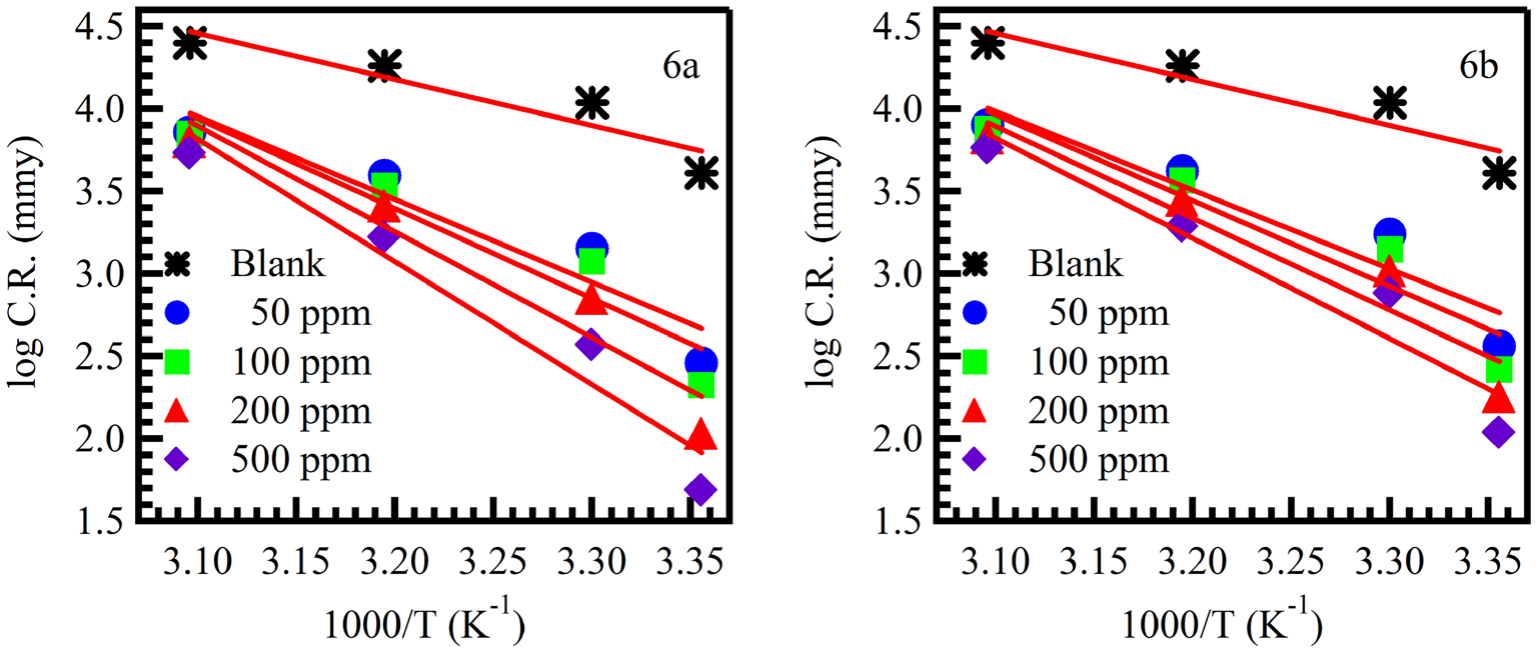
Arrhenius plots for mild steel in 1 M HCl solution without and with different concentrations of the inhibitors.

The Apparent activation energies (− Ea/2.303R) and pre-exponential factor (A) at different concentrations of (6a and 6b) are determined by linear regression between Log (CR) and 1/T Fig. 7. The activation energy values in the absence and in the presence of inhibitors at different temperatures are reported in (Table 3)^56,57^. In the presence of the inhibitor the values of E_a_ are higher than in its absence. This behavior is reported as being characteristic of a phenomenon of physisorption of the inhibitor on the surface of the metal. The recovery rate, which is very low at higher temperatures high, suggests that at these temperatures the rate of destruction of the physically adsorbed film increases faster than its formation rate. This phenomenon can also be explained by the fact that the corrosion process of the mild steel in the presence of the inhibitor does not depend only on the reaction that takes place on the surface of the bare metal, but also from the diffusion of ions iron through the adsorbed inhibitor layer. This confirms that the inhibitors at high concentrations participate in a stronger physical adsorption by the formation of a more adherent surface film and therefore more effective^58,59^.

The values of kinetic parameters such as enthalpy (ΔH) and entropy (ΔS) of corrosion process may be evaluated from the temperature effect on MS surface were obtained using Eq. (12)^58^.

12 $$\mathrm{CR}=\frac{\mathrm{RT}}{{\mathrm{N}}_{\mathrm{A}}\mathrm{h}}\mathrm{exp}\left(\frac{\mathrm{\Delta S}}{\mathrm{R}}\right)\mathrm{exp}\left(-\frac{\mathrm{\Delta H}}{\mathrm{RT}}\right),$$

where, h is Planck’s constant, NA is Avogadro’s number.

**Figure 8:**
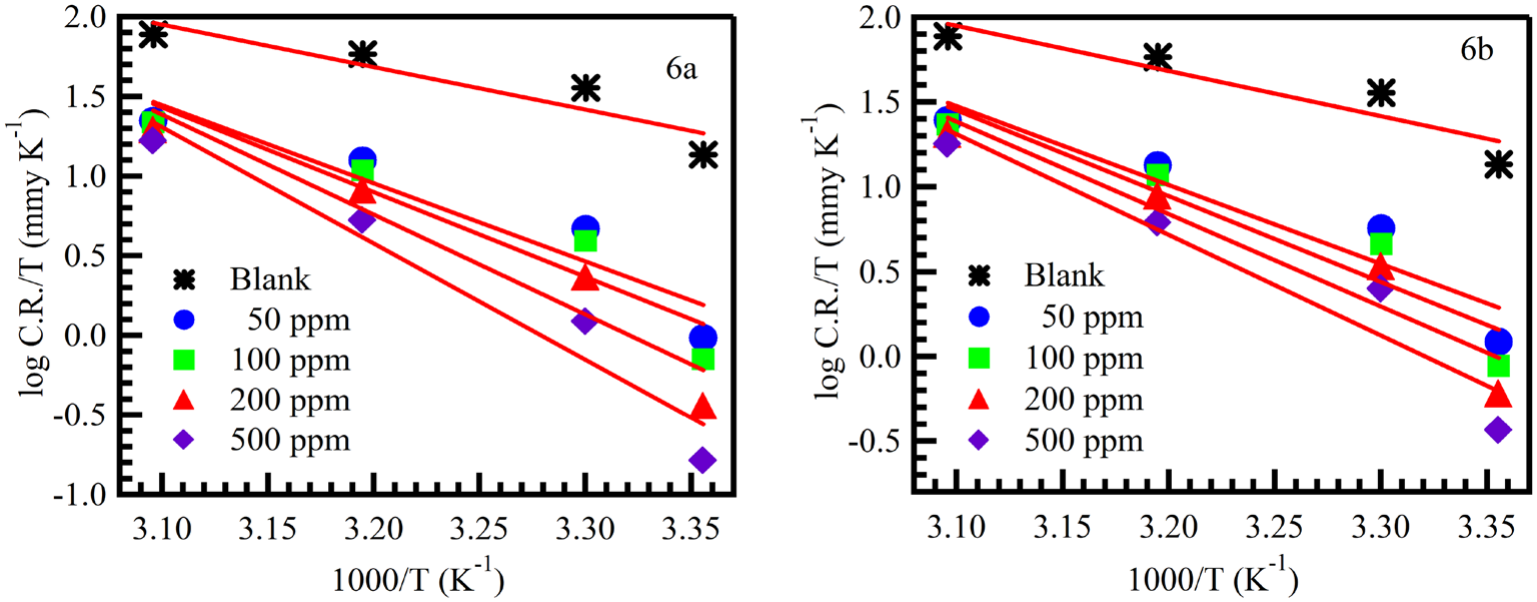
Transition state plots for mild steel dissolution in the absence and presence of the inhibitors.

Enthalpy and entropy values of (6a and 6b) compounds on MS surface were calculated using the transition state Eq. (5). Figure 8 shows log (CR/T) against 1000/T, which give straight lines with a slope of − ∆H/2.303R and an intercept of [log(R/NAh) + (∆S/2.303R)] for MS in the absence and presence of (6a and 6b) compounds have concentration 50, 100, 200 and 500 ppm in 1 M HCl solution.

In (Table 3) Values of ΔH* were found positive. Positive values indicate endothermic nature of mild steel dissolution process^51,60^. Endothermic process further indicates that mild steel dissolution reduces at lower temperatures and increases with increase in temperatures. Negative values of ΔS* are indicative of formation of activated complex in rate determining step, which represents association rather than dissociation step, meaning the decrease in disorder takes place on going from reactants to activated complex^61,62^.

### Electrochemical study

**Figure 9:**
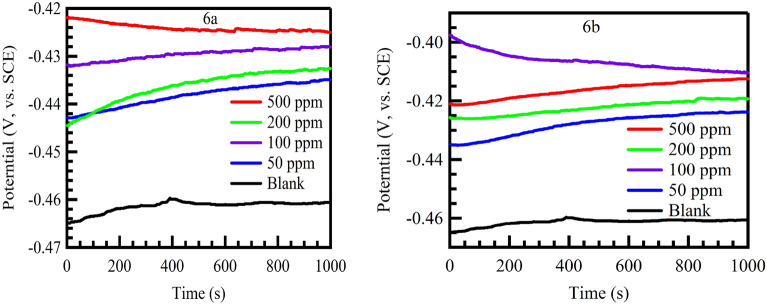
Open circuit potential plots for mild steel in 1 M HCl in absence and presence of different concentration of inhibitors 6a and 6b at 298 K.

Open circuit potential (OCP) curves for the corrosion of carbon steel in 1 M HCl in absence and presence of different concentrations of investigated inhibitors (VI, VII and VIII) at 298 K are shown in Fig. 9.

**Figure 10:**
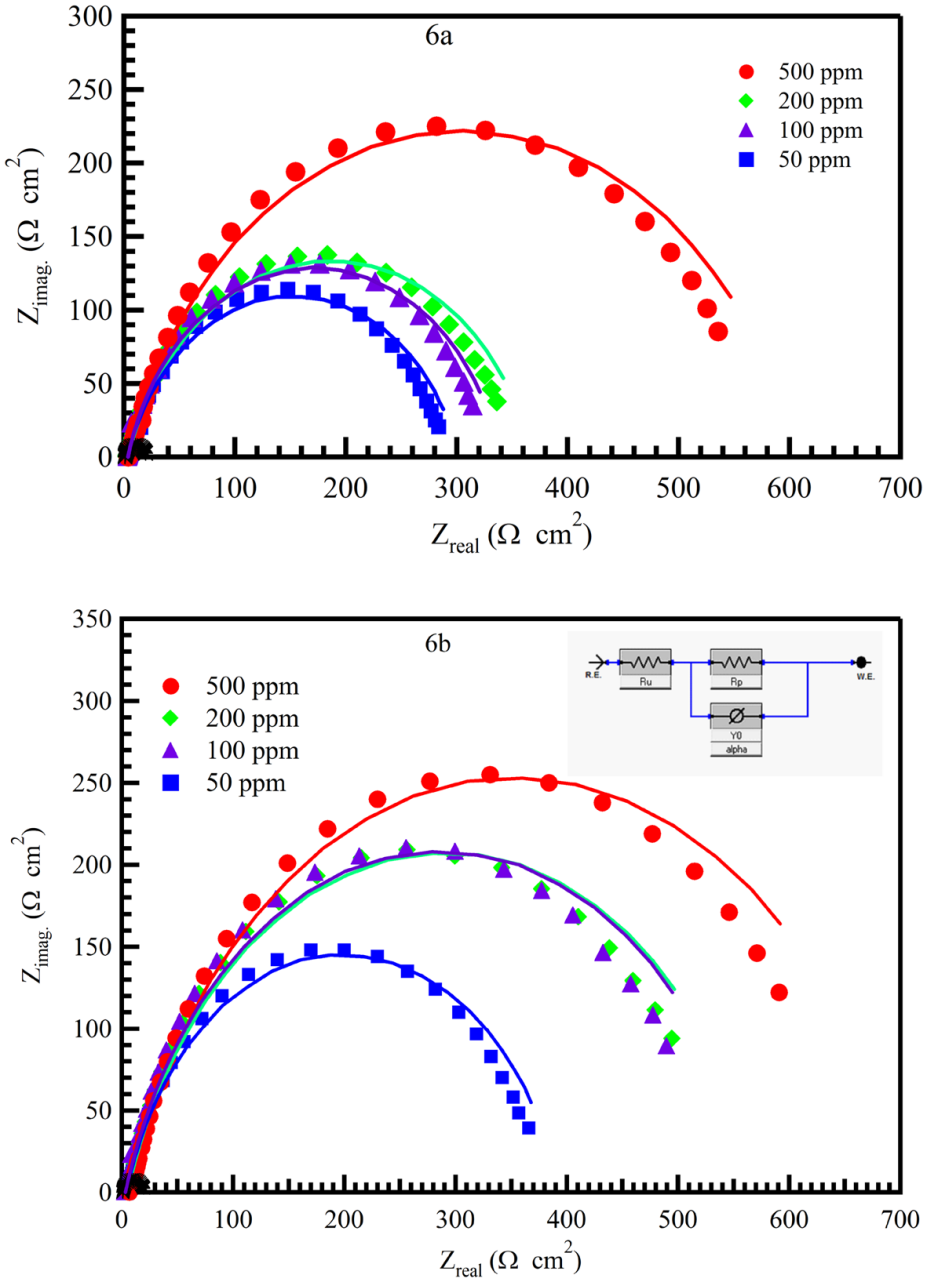
Nyquist plots for mild steel in 1 M HCl in absence and presence of different concentration of inhibitors 6a and 6b at 298 K (the inset shows the equivalent circuit model used to fit the EIS data, and the solid line is the fitting data).

Measurements using electrochemical impedance spectroscopy (EIS) are a valuable technique for characterizing a variety of electrochemical systems and comprehending the function of electrolytic processes, such as batteries, and the behavior of elements during corrosion. Figure 10 shows the Nyquist plots for MS at different concentrations for the investigation of corrosion.

The capacitance loops show that mild steel corrosion and the formation of a surface barrier are predominantly controlled by the electron transfer process^63^. The equivalent circuit model, shown in Fig. 10, was used to fit the experimental data on mild steel’s impedance in the presence of inhibitors in 1M HCl. The solution resistance in this Figure is denoted by Rs (Ru), the charge transfer resistance is denoted by Rct (Rp), and the double-layer capacitance on the metal’s surface is denoted by (Cdl)^36^. Table 4 shows that the addition of an additive to a solution of 1 M HCl causes the R_ct_ values for MS to increase, indicating that the charge transfer pathways are hindered.

**Table 4:** Electrochemical impedance spectroscopy parameters of MS in 1 M HCl in the absence and presence of different concentrations of 6a and 6b at 298 K.

Inhibitors	Conc (ppm)	Rs (Ω cm^2^)	Rct (Ω cm^2^)	Y0 (Ω^−1^ s^n^ cm^−2^)	n	Cdl (µF cm^−2^)	θ	IE EIS %
Blank	0.0	0.5816	20.34	723.80	0.903	461.02	–	–
6a	50	2.474	244.6	419	0.841	272.88	0.92	91.68
	100	0.619	267.9	284	0.872	194.66	0.92	92.41
	200	0.787	334.8	160	0.758	62.88	0.94	93.92
	500	1.038	391.6	94	0.678	19.45	0.95	94.81
6b	50	4.35	299.6	344	0.996	341.39	0.932	93.21
	100	3.37	335.9	369	0.933	318.52	0.939	93.94
	200	5.25	365.8	399	0.855	288.20	0.944	94.44
	500	7.17	490.6	309	0.795	190.37	0.959	95.85

Inhibition efficiency (IE_(EIS)_) and θ were calculated from the following Eqs. (13) and (14)^46^:

13 $${\mathrm{IE}}_{\left(\mathrm{EIS}\right)}=\uptheta \times 100=\left[\frac{{\mathrm{R}}_{\mathrm{ct}\left(\mathrm{inh}\right)}-{\mathrm{R}}_{\mathrm{ct}\left(\mathrm{unh}\right)}}{{\mathrm{R}}_{\mathrm{ct}\left(\mathrm{inh}\right)}}\right]\times 100,$$

where R_ct(inh)_ and R_ct(unh)_ are charge transfer resistances in the presence and absence of an inhibitor, respectively

14 $$\mathrm{Cdl }= {\left({\mathrm{Y}}_{0}{\mathrm{ Rct }}^{1-\mathrm{n}}\right)}^{\frac{1}{\mathrm{n}}},$$

where the CPE exponent is n and Y_0_ is the CPE constant. The number n, which ranges from 0 to 1, represents the departure from the ideal behavior.

When the inhibitor was applied, the value of C_dl_ in Table 4 decreased, indicating a decrease in the local dielectric constant and/or an increase in the thickness of the electrical double layer, which suggests that the inhibitor molecules work by forming a protective layer at the metal surface^64^.

EFM is an electrochemical method for calculating the corrosion rate without prior knowledge of the Tafel constants. This method’s ability to assess the corrosion rate, Tafel parameters, and causative factors all in one data set. Table 5 provides the corrosion parameters for protection effectiveness, corrosion current density, Tafel constant, and causative factors (CF-2), (CF-3) for various concentrations in 1M HCl at 298 K.

**Table 5:** EFM parameters of MS in 1 M HCl in the absence and presence of different concentrations of 6a and 6b at 298 K.

Inhibitor	C	Icorr	βa	βc	K	CF (2)	CF (3)	θ	IE EFM%
	(ppm)	(uA)	(mV dec^−1^)	(mV dec^−1^)	(mpy)				
Blank	–	1007	73.76	92.82	460.30	2.033	3.191	–	–
**6a**	50	83.9	207	212	38.360	1.575	1.865	0.917	91.668
	100	76	45.41	48.00	34.730	1.474	2.332	0.925	92.457
	200	61.2	30.20	30.83	27.988	1.016	2.91	0.939	93.923
	500	51.9	16.68	17.39	23.743	1.49	3.08	0.948	94.845
**6b**	50	71.4	42.57	45.97	33.08	1.09	2.622	0.929	92.91
	100	68.4	44.96	48.67	30.70	1.8	2.544	0.932	93.21
	200	57.7	36.96	39.79	26.37	1.15	2.434	0.943	94.27
	500	44.7	22.17	22.77	20.40	2.6	3.371	0.956	95.56

**Figure 11:**
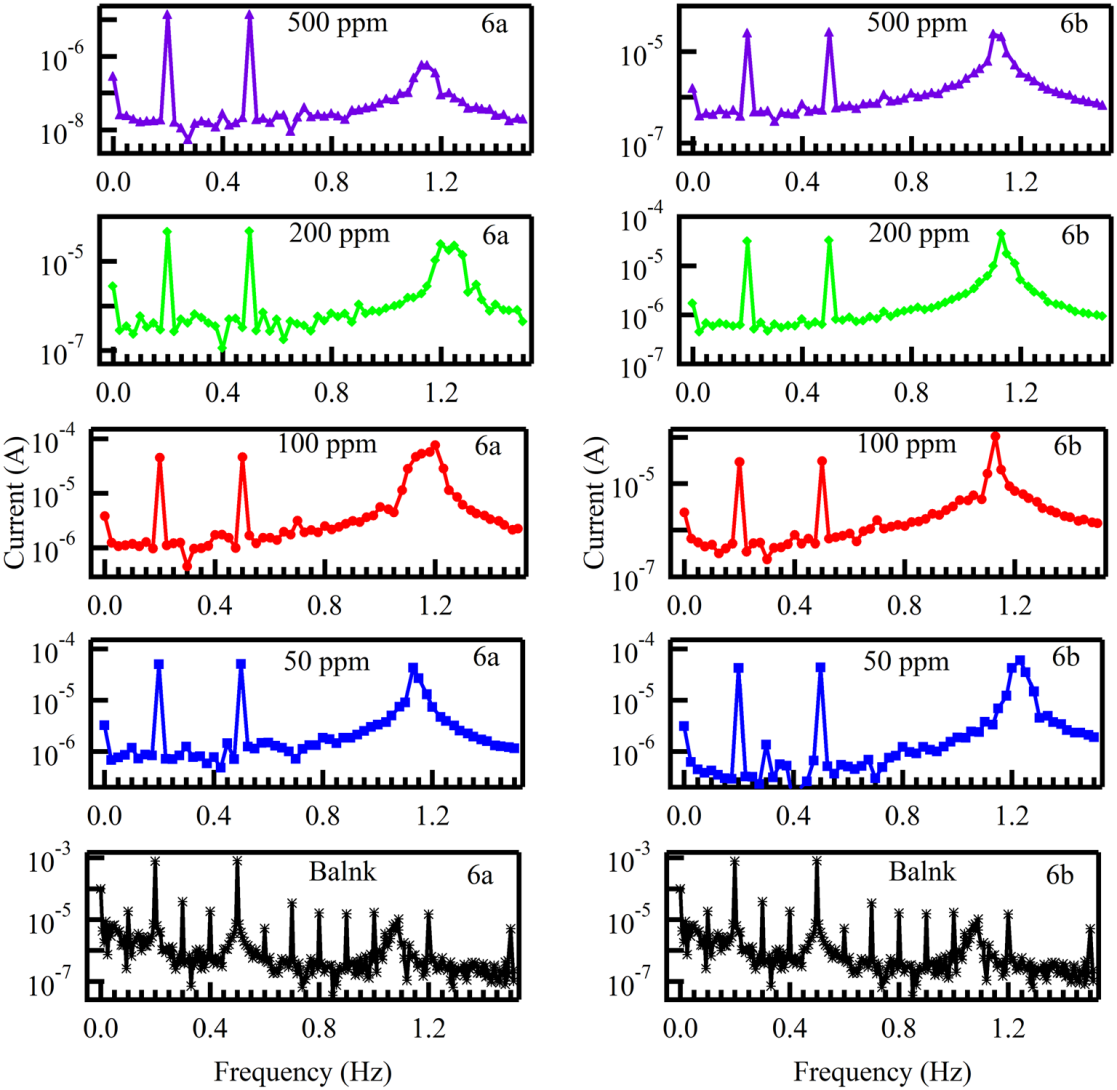
Intermodulation spectrums for the corrosion of in 1 M HCl solution at different inhibitor concentrations at 298 K.

Examples of the EFM intermodulation spectra of mild steel in 1 M HCl solutions in the absence and presence of various concentrations of the inhibitors 6a and 6b are shown in Fig. 11 at 298 K. Equation can be used to compute the surface coverage and the inhibition efficiency (IE_EFM_%)^65^:

15 $$\mathrm{\%IE}\left(\mathrm{EFM}\right)=\uptheta \times 100=\left(1-\frac{\mathrm{Icorr }\left(\mathrm{inhibitors}\right)}{\mathrm{Icorr }\left(\mathrm{blank}\right)}\right)\times 100,$$

where Icorr (inhibitors) and Icorr (blank) are corrosion current densities.

This Table 5 clearly demonstrates that as the concentration of inhibitors increases, the values of I_corr_ decrease, indicating that (IE_EFM_) increases, indicating that the inhibitors prevent corrosion by adsorption on the MS surface by forming chemical and physical bonds and creating a barrier of protection that lowers the ratio of corrosion^66^.

According to the EFM theory, the values of the causality factors (CF-2, CF-3) are very similar to their theoretical values (2) and (3), indicating that Tafel slopes and corrosion current densities are correct^67^.

**Figure 12:**
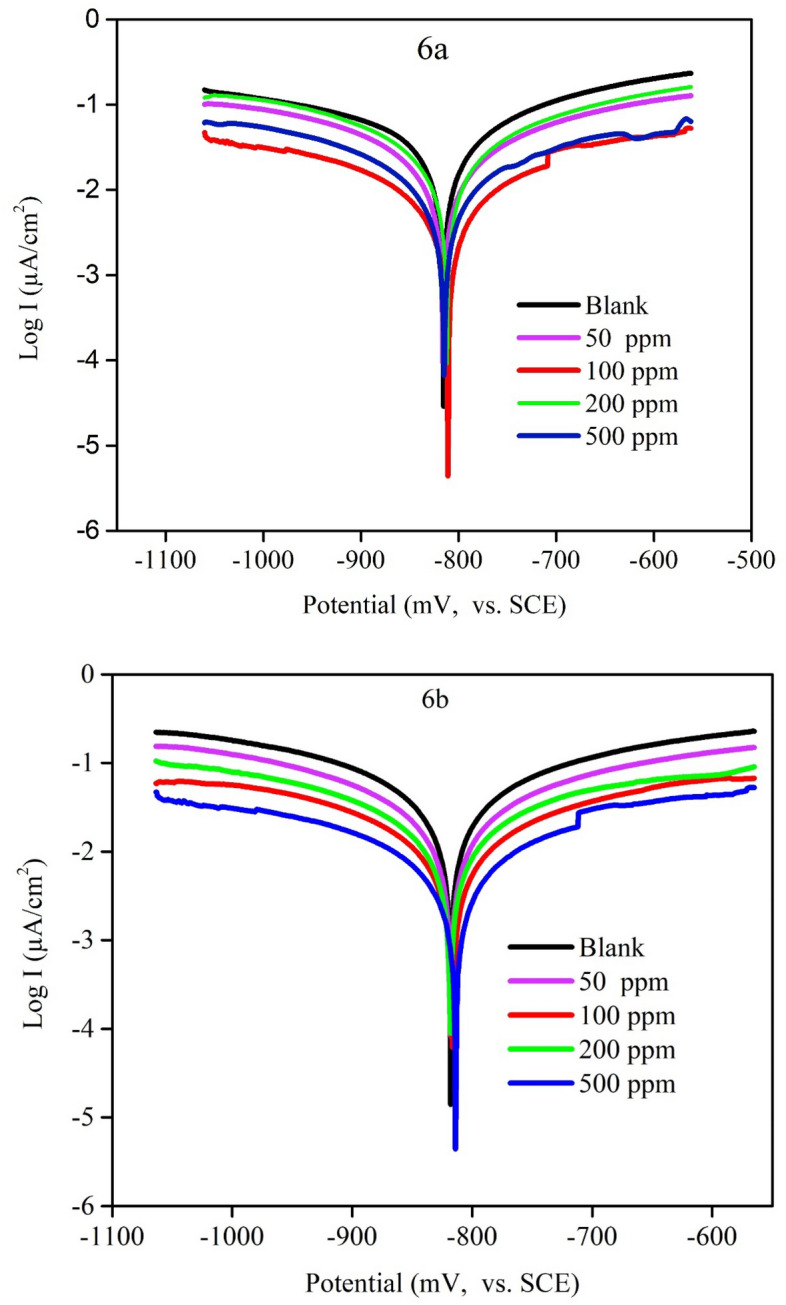
Tafel plots for MS in 1 M HCl solution at different inhibitors concentrations at 298 K.

Polarization curves of MS in 1 M HCl solution at 298 K with different concentrations of the inhibitors are shown in Fig. 12. The percentage inhibition efficiency (IE_PDP_%) and the degree of surface coverage (θ), were calculated from Eq. (16)^68^.

16 $$\mathrm{\%IE}\left(\mathrm{PDP}\right)=\uptheta \times 100=\left(1-\frac{\mathrm{icoor }\left(\mathrm{inhibition}\right)}{\mathrm{icoor }\left(\mathrm{free}\right)}\right)\times 100.$$

The plots of potential versus the logarithm of current density were used to display polarization curves. Table 6 includes a list of the corrosion characteristics, including corrosion potential (Ecorr), corrosion current density (Icorr), anodic and cathodic Tafel slopes (a and c), and percentage inhibition efficiency (IE_PDP_%). It is so clear that Icorr of anodic and cathodic reactions reduced with increasing the concentration of the inhibitor till reach the maximum that guarantee the inhibition process.

**Table 6:** Corrosion parameters obtained from potentiodynamic polarization measurements of mild steel in 1 M HCl at different inhibitor concentrations 6a and 6b at 298 K.

Inhibitor	C (ppm)	Βa (mV dec^−1^)	Βc (mV dec^−1^)	Icorr (uA)	E_corr_ vs. SCE (mV)	K (mpy)	Chi squared	θ	IE PDP%
Blank	–	313	184	4180	− 339	1911.000	85.36	–	–
6a	50	203	275	410	− 419	187.400	49.58	0.902	90.191
	100	208	298	300	− 428	136.900	77.47	0.928	92.823
	200	205	307	259	− 425	118.100	77.53	0.938	93.804
	500	222	296	224	− 427	102.400	94.08	0.946	94.641
6b	50	350	263	276	− 424	126.100	111.2	0.934	93.397
	100	323	230	250	− 424	113.400	86.81	0.940	94.019
	200	317	270	177	− 418	80.710	92.45	0.958	95.766
	500	318	260	140	− 413	63.740	58.68	0.967	96.651

This barrier of defense is created on the surface of the mild steel by the heteroatoms and unsaturated bonds in the inhibitor. Based on the sort of reaction that often takes place in an acidic solution, an inhibitor can be categorized as either an anodic, cathodic, or mixed type inhibitor. The cathodic process, also known as the release of hydrogen gas, is the opposite of the anodic reaction, which involves the dissolving of metal and releasing electrons^69^.

### Scanning electron microscopy

**Figure 13:**
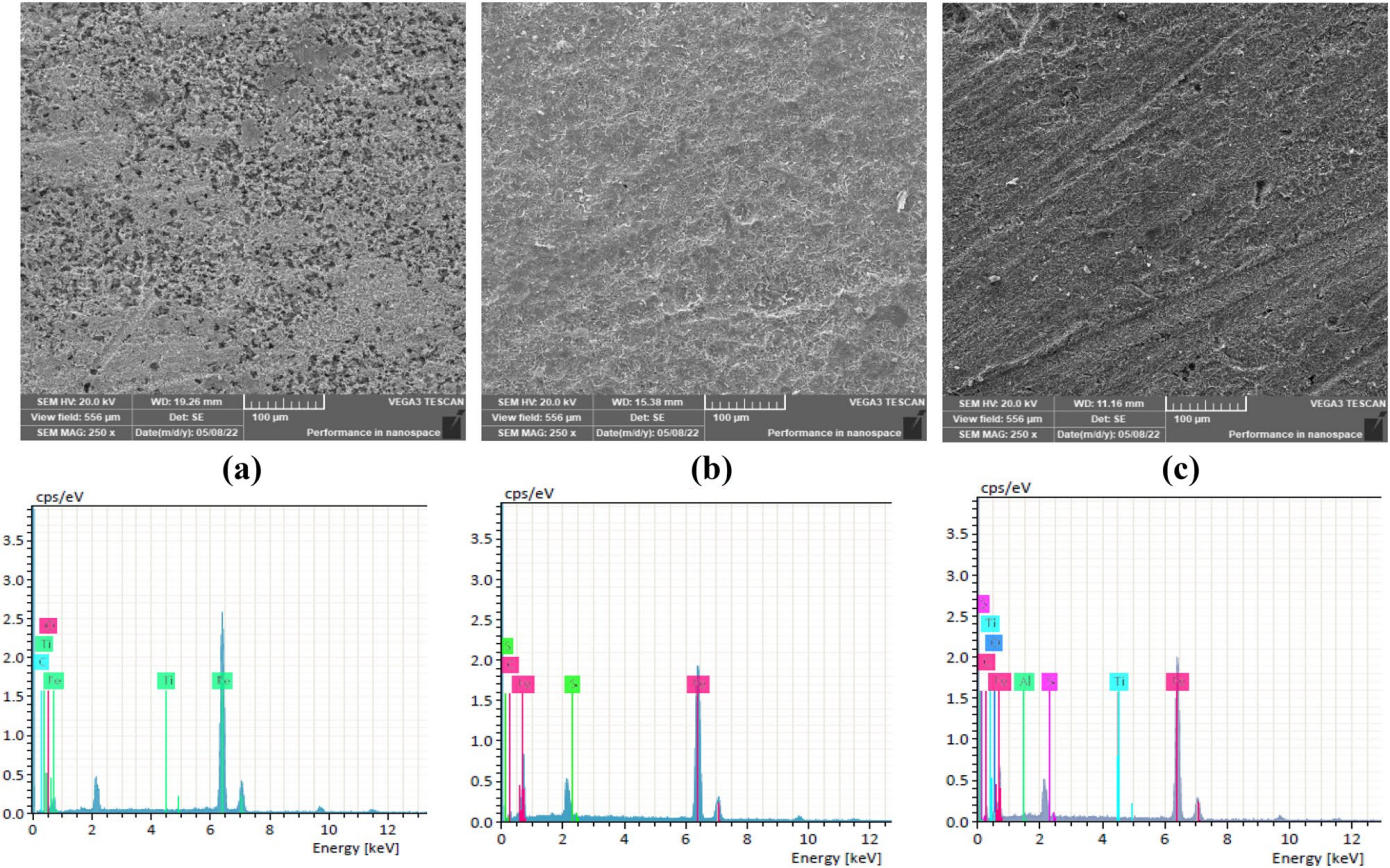
SEM images and EDX of MS surface after 1 day immersed in 1 M HCl (**a**) blank, (**b**) 6a, and (**c**) 6b at 298 K.

The polished MS surface is extensive in the case of the MS without corrosion inhibitors seen in Fig. 13a, and this might be interpreted as severe surface damage. The surface smoothed out while the inhibitors 6a and 6b were present (Fig. 13b,c). This shows that the inhibitors adhered to the MS surface and created a layer of protection. These results are supported by EDX analysis, which reveals that the main components are just Fe, Ti, and O in the MS itself. While both S and C are present in the MS with 6a and 6b as inhibitors, those from 6a and 6b are the ones that are original.

### Quantum chemical calculations

Many factors affect how organic molecules inhibit metal corrosion in an acidic medium^70^ such as: the number of adsorption centers, the mode of interaction with the metal surface, the size and the molecular structure^71^ and in order to give more interpretations to the experimental results, different quantum parameters namely: EHOMO, ELUMO, ΔE = EHOMO − ELUMO, (σ), (η), (ω), (µ, Debye) and (ΔN). E_HOMO_ (the energy of the first unoccupied molecular orbitalis commonly related to the molecule’s ability to transfer electrons. A high level of HOMO energy encourages a molecule’s ability to donate electrons to species possessing unfilled molecular orbitals whose energy level is low. On the other hand, ELUMO (the energy of the highest occupied molecular orbital) reflects the molecule’s ability to receive electrons. A low value of ELUMO represents that the molecule accepts electrons.

ΔE = EHOMO − ELUMO is the least energy required to excite an electron in a molecule. Therefore, a low value of ΔE leads in a considerable inhibitory effectiveness^72–75^. Table 7 gather the calculated quantum parameters for the 6a and 6b compounds. This table shows that, molecules with a low energy gap have better inhibitory efficiency, this is explained by the fact that these compounds readily move one or more electrons from the HOMO level to the empty “d” orbitals of iron, and thus enhancing the sharing of electrons between these molecules and the surface of the metal^52,76^. Furthermore, according to the literature, a rise in EHOMO values can speed up adsorption by affecting how species move across the adsorbed layer^76^. In Table 7, It was evident from the results that 6b is higher to 6a in terms of effectiveness.

**Table 7:** The calculated theoretical chemical parameters of MPBS at B3LYP/6-311G (d,p) basis set method.

Method	DFT/B3LYP/6–311 G (d,p)								
Comp.	Parameters								
	E_H_ (ev)	E_L_ (ev)	E∆ (ev)	η (ev)	σ (ev)	ω (ev)	Dm (Debye)	T.N.C	N∆ (ev)
6a	− 8.3198	− 5.855	2.4645	1.2323	0.8115	40.224	6.0183	− 4.3539	0.0361
6b	− 8.0093	− 5.856	2.1538	1.0769	0.9286	44.628	6.6146	− 5.2936	0.0314

A high absolute hardness value is a sign of a molecule’s excellent stability and low reactivity. Table 7 revealed that the η.p % values for the examined inhibitors drop as the hardness values rise. 6a is the hardest of the inhibitors (η = 1.2323). The inhibitor 6b has lowest hardness (η = 1.0769). The values of increasing hardness are in good agreement with the experimental IE % values.

The nucleophilic or electrophilic character of the molecule is shown by the electrophilicity index (ω) readings. In contrast, a low value of electrophilicity indicates that the molecule has a high tendency to function as a nucleophile. A high electrophilic value indicates that the molecule has a strong inclination to behave as an electrophile. A good electrophile is defined by a high value of ω and an excellent nucleophile is associated with a lower value of ω^77^. From the Table 7 the electrophilicity values increase as follows: (44.628 > 40.224 eV) with comparing electrophilicity index values with the IE exp% (95.17 > 94.02 at 298 K) for (6a and 6b) compounds, respectively.

Depending on the kind and nature of the molecules involved, the inhibitory effectiveness rises as the dipole moment value grows^78^. Table 7 illustrated that when the values of µ grew, so did the values of IE%. The highest value applies to the inhibitor 6b (6.6146 D). The lowest value for the inhibitor is 6a (6.0183D). By Lukovits’s study, if the value of ΔN < 3.6, the η.p % increase by increasing electron donating ability of inhibitor on the metal surface^79–81^. From Table 7, generally, values of ∆N range from 0.0314 to 0.0361. 6b indicates the highest value transfer of electron and hence greater IE%. Thus, the fraction of transmitted electrons is the largest for 6b as compared with other inhibitor 6a.

The Mullikan population analysis is employed to calculate the charge distribution across the entire skeleton of the molecule and to determine the adsorption sites of inhibitors^82,83^. The calculated Mulliken charges (Total negative charges) of selected atoms carbon, oxygen and nitrogen were presented in Table 7. It is predicted that 6a will contact the metal surface more often but 6a is expected to do so less.

**Figure 14:**
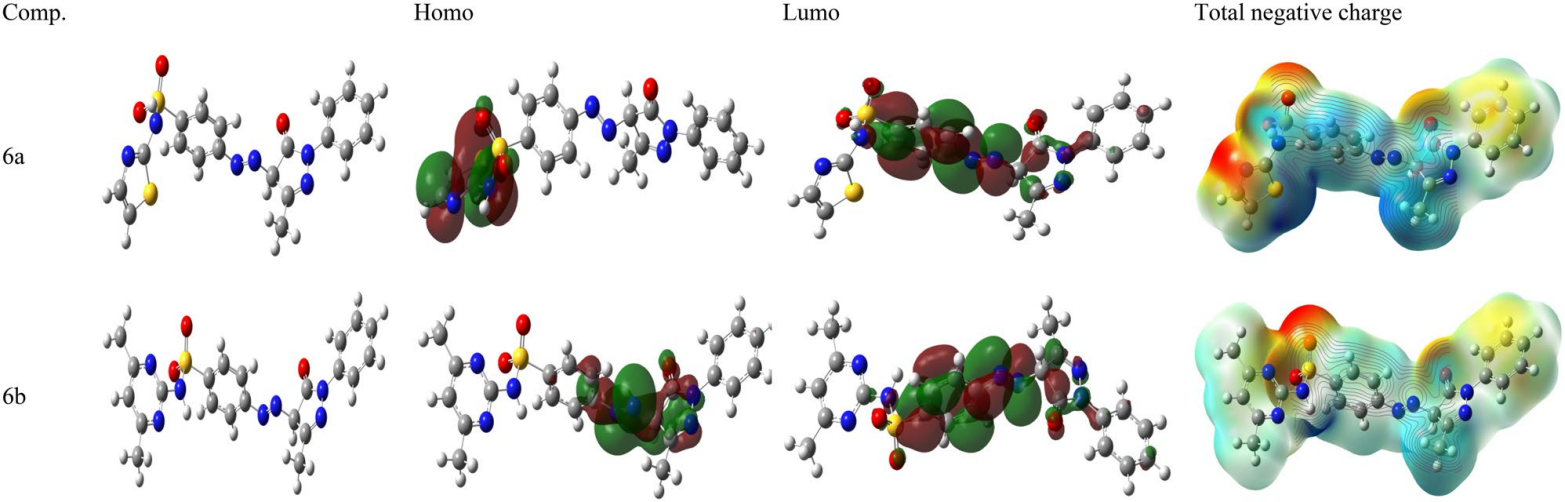
(**a**) The optimized molecular structure, homo, lumo and, total negative charge of the investigated 6a and 6b using (DFT) 6-311G (d,p) basis set method.

The distribution of the HOMO and LUMO electron density of the molecules are represented in Fig. 14 We thus observe for all the inhibitory molecules, the distribution of the HOMO density is centered on the heteroatoms (S, N and o) of the pyrazol ring and benzene sulfonamide. The high inhibitory power of benzene sulfonamide can be attributed to the presence of aromatic substituents possessing “π” electrons encouraging the sharing of electrons between these compounds and the metal surface. In addition, the concentration of anions on the metal surface will enhance the adsorption of the cationic forms of the benzene sulfonamide inhibitors^34^. The nearest area near the sulphur atom has the greatest HOMO density values, which is concrete proof that sulphur is the nucleophilic center^84–86^. As a result, sulphur will readily establish the metal–metal connection rather than N or C atoms. Table 8 compares pyrazolone-sulfonamide hybrids inhibition activity with other related inhibitors^36,87–90^.

**Table 8:** Comparison of the inhibition efficiency of pyrazolone-sulfonamide hybrids inhibitors with other related inhibitors.

Inhibitor	HCl concentration	IE%	Ref.
4-Amino-*N*-carbamimidoylbenzenesulfonamide	3%	97.6	^1^
*N*-(4,6-Dimethylpyrimidin-2-yl)-4-(quinazolin-4-ylamino)benzenesulfonamide	1 M	97.6	^2^
4-Amino-*N*-(pyrimidin-2-yl)benzenesulfonamide	1 M	94.0	^3^
4-Amino-*N*-(5-methylisoxazol-3-yl)benzenesulfonamide	1 M	93.2	^3^
4-Amino-*N*-(thiazol-2-yl)benzenesulfonamide	0.5 M	63.1	^4^
(*E*)-4-((3-Methyl-5-oxo-4,5-dihydro-1H-pyrazol-4-yl)diazenyl)benzenesulfonamide	1 M	95.4	^5^
(*E*)-4-((3-Methyl-5-oxo-1-phenyl-4,5-dihydro-1H-pyrazol-4-yl)diazenyl)-*N*-(thiazol-2-yl)benzenesulfonamide	1 M	96.6	This study

## Conclusions

In this study two compounds successfully synthesized and characterized FTIR, ^1^H-NMR, ^13^C-NMR, and mass spectra. It was then used as inhibitor corrosion of mild steel in 1 M HCl by using the gravimetric method, electrochemical measurement, scanning electronic microscope analysis, and quantum chemical calculations. The studied chemicals’ inhibitory potency ranged from 76.99 to 96.651%. These compounds attach to the Langmuir adsorption isotherm during the adsorption process. A few isotherms and kinetic parameters for adsorption have been created and explored. The mechanism of inhibition is mixed type. The metal surface had a thin protective layer that acted as an inhibitor, according to analyses done with energy dispersive X-ray spectroscopy (EDX) and scanning electron microscopy (SEM).

### Supplementary Information

Supplementary Figures.

## Data Availability

All data generated or analyzed during this study are included in this published article (and its Supplementary Information files).
