# Supplementary material for: Corrosion inhibition of mild steel in 1 M HCl by pyrazolone-sulfonamide hybrids: synthesis, characterization, and evaluation
Source: Sci Rep. 2023 Oct 29;13:18555. doi: 10.1038/s41598-023-45659-2 (PMC10613630; doi:10.1038/s41598-023-45659-2)
Supplement: Supplementary file 1 — Supplementary Figures. [file 41598_2023_45659_MOESM1_ESM.docx]

a

The following tautomeric isomers are possible for l-phenyl-3-methyl-5-pyrazolones:


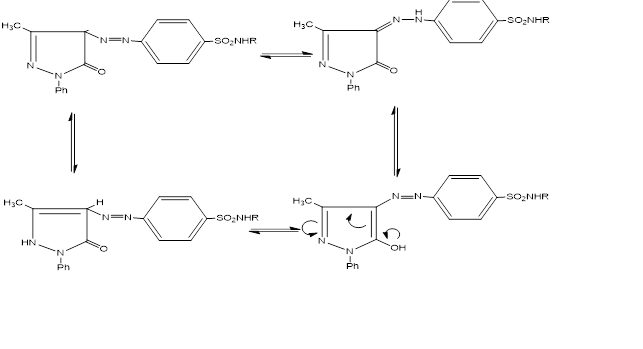


b

Figure S1. **a**) Proposed mechanism for synthesis of pyrazolone-sulfonamide hydrids**6_a,b._ b)** Tautomeric forms of pyrazolone-sulfonamide hydrides **6_a,b_**


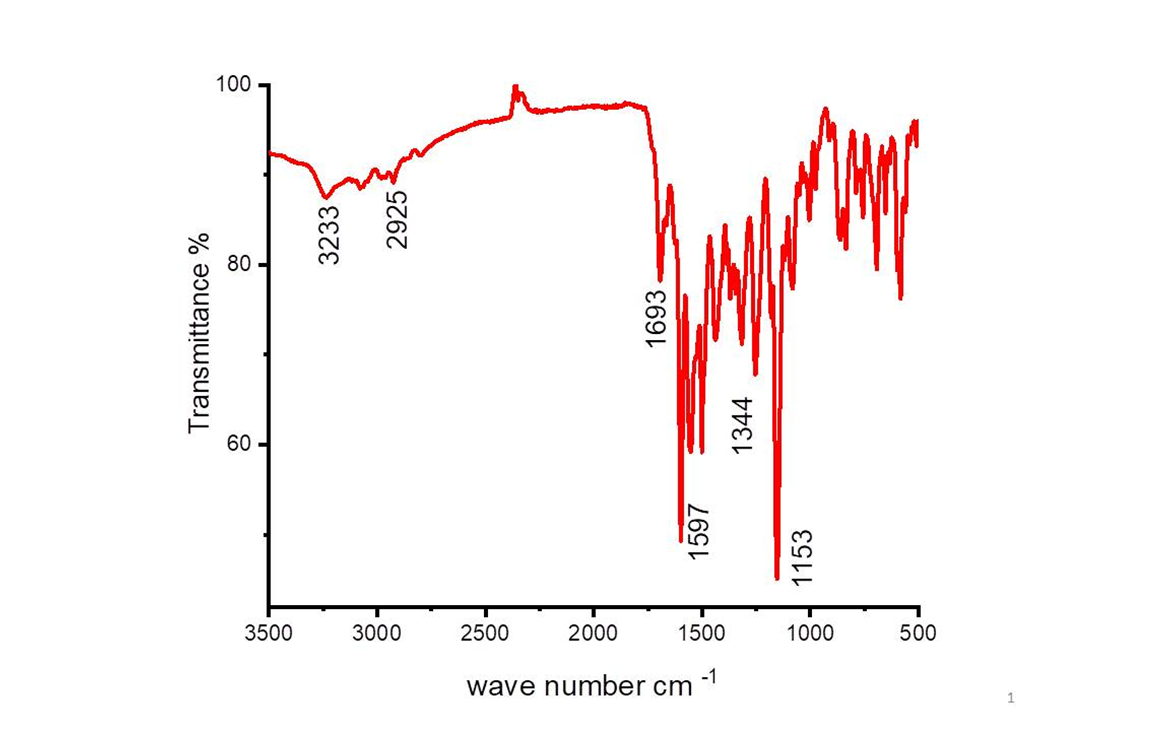


a


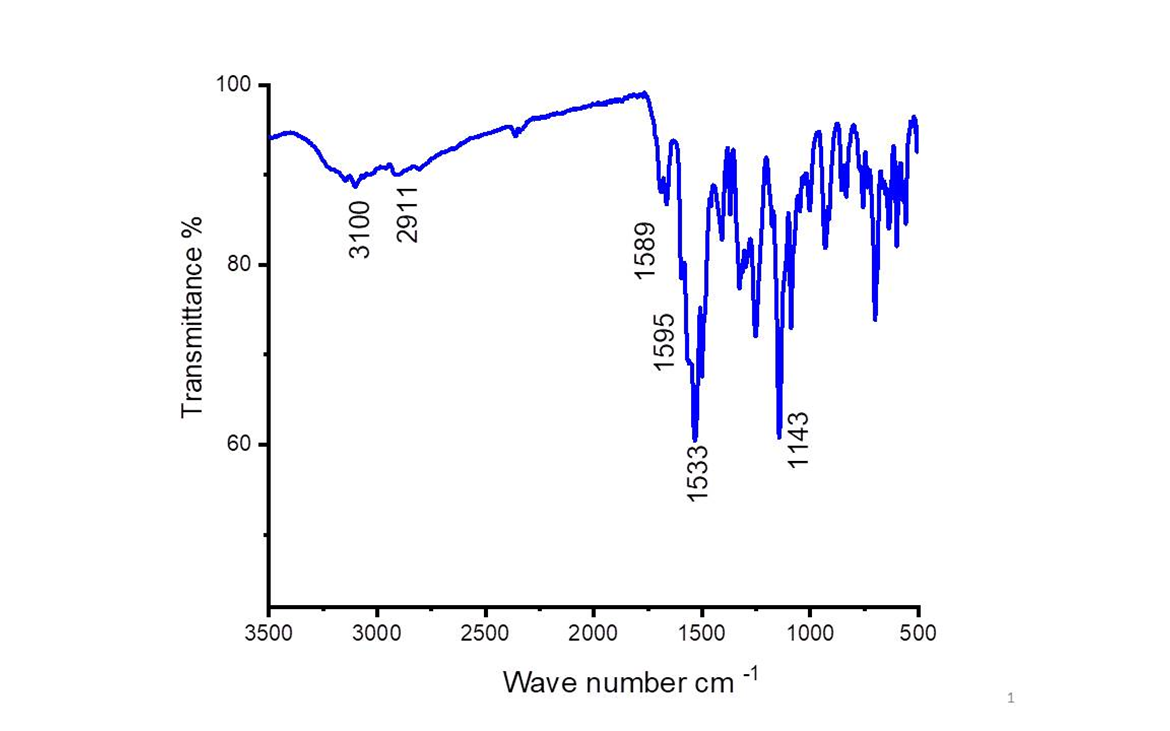
 Figure S2. FTIR of the synthesized compound a)6a and b)6b

b
